# Proteome of the testicular cell-conditioned medium supports germ cell differentiation *in vitro*

**DOI:** 10.5455/javar.2025.1969

**Published:** 2025-12-25

**Authors:** Wahono Esthi Prasetyaningtyas, Ni Wayan Kurniani Karja, Srihadi Agungpriyono, I Ketut Mudite Adnyane, Kusdiantoro Mohamad, Mokhamad Fahrudin

**Affiliations:** 1 Division of Anatomy, Histology, and Embryology, School of Veterinary Medicine and Biomedical Sciences, IPB University, Bogor, Indonesia; 2 Divisions of Reproduction and Obstetrics, School of Veterinary Medicine and Biomedical Sciences, IPB University, Bogor, Indonesia

**Keywords:** Conditioned medium, germ cells, *in vitro* spermatogenesis, proteomics, testicular cells

## Abstract

**Objective:**

This study aimed to evaluate the effect of testicular cell conditioned medium (TCCM) on *in vitro* male germ cell differentiation and provide a proteomic profile of TCCM.

**Materials and Methods:**

TCCM was collected from 5-day-old mouse testicular tissues cultured in serum-free DMEM. Proteomic analysis was performed using liquid chromatography–tandem mass spectrometry. Germ cells were isolated from 13.5 days post-coitum (dpc) mouse fetal genital ridges and divided into three groups: (a) control (DMEM + 15% FCS), (b) 40% TCCM + 60% DMEM + 15% FCS, and (c) 60% TCCM + 40% DMEM + 15% FCS. Cells were cultured for 24 days. Gene expression of *Oct4*, *Acr*, *Dazl*, *Vasa*, *Stra8*, *Prm1*, and *Gdf9* was measured using real-time PCR.

**Results:**

Proteomic analysis identified 26 proteins in TCCM. Notably, COL4A1, COL4A2, and HSPG2 are associated with the basement membrane and are essential for supporting extracellular matrix integrity, while FN1, FBN1, COL1A1, COL1A2, COL5A2, and COL3A12 are linked to the PI3K-AKT pathway, which regulates cell proliferation. In TCCM-treated groups, germ cells differentiated into spermatid-like cells by day 18 and sperm-like structures by day 24. *Oct4*, *Dazl*, *Vasa*, *Stra8*, *Prm1*, and *Acr* were expressed across all groups, but without statistically significant differences (*p* > 0.05), while *Gdf9* was not expressed.

**Conclusion:**

The addition of TCCM, which contains extracellular matrix (ECM) proteins, enhanced *in vitro* differentiation of male germ cells into sperm-like structures, together with somatic cells from the genital ridge. These ECM proteins contribute to creating a microenvironment that closely mimics *in vivo* conditions.

## Introduction

Infertility is a significant issue with high emotional and psychological costs to communities and families. Male infertility can be caused by a variety of factors, most notably azoospermia. Furthermore, several factors, including genetics, treatments, and trauma, contribute to the loss of germ cells from the testes [1]. There are many methods to treat male infertility, including assisted reproductive technology [2], *in vitro* spermatogenesis [3,4], lifestyle changes, and medical treatment (gonadal therapy) .

Primordial germ cells are totipotent precursors of male and female gamete cells [6–10]. Totipotency causes primordial germ cells (PGCs) to share similarities with embryonic stem cells, including their morphology, development, and genetics [11,12], which makes PGCs potentially useful as a therapy for infertility [10,13]. PGCs originate from the epiblast on embryonic day six. The cells can be observed in the allantois and begin to migrate at E 7-8. The PGCs are then integrated into the epithelium of the hindgut, and at E9-10, mPGCs start to migrate into the dorsal mesentery, reaching E10-11 [3]. PGCs in females continue to proliferate to become oogonia and then enter meiosis to become primary oocytes at approximately E13.5. In contrast, PGCs enter mitotic arrest in males after substantial proliferation to become gonocytes from approximately E15.5 [4]. Previous research has highlighted various applications of PGCs, including those used for the conservation and production of hybrid poultry [6]. Furthermore, PGCs have been reported to aid in treating infertility, particularly azoospermia in mice [7]. Transplantation of PGCs into the testes has been reported to successfully develop into functional gametes [8] or into functional spermatozoa and oocytes after transplantation into the renal subcapsular [9].

Owing to the limited availability of PGCs in the genital ridge, a method to multiply these cells is necessary, typically through *in vitro* culture. However, PGCs often differentiate in culture, necessitating the development of appropriate culture methods that allow for their proliferation without losing their totipotent properties [10]. Observing PGC growth in an *in vitro* medium could help identify factors that influence PGC proliferation and differentiation, which may be related to clinical cases. It is believed that PGCs differentiate into gonocytes by day three of culture and reach the leptotene stage after five days [11]. Prasetyaningtyas et al. [12] reported that culturing PGCs for 6 days with the addition of 1,000 IU/ml leukemia inhibitory factor (LIF) increased the number of cells expressing *Oct4*. This increase suggests that PGCs may have differentiated into spermatogonial stem cells (SSCs) by day six. PGCs and SSCs express *Oct4*, indicating their pluripotent nature [13,14].

The development of PGCs *in vivo* and *in vitro* requires environmental signals to facilitate mitosis and meiosis, ultimately leading to differentiation of male and female germ cells [7]. *In vitro*, PGC can enter meiosis when exposed to niches rich in growth factors, such as those derived from testicular tissues [11]. These niches indicate that both somatic and spermatogenic cells produce essential growth factors for spermatogenesis, including BMP4, stem cell factor (SCF), LIF, basic fibroblast growth factor, retinoic acid, insulin-like growth factor 1 (IGF-1), and glial cell-derived neurotrophic factor (GDNF) in the testes [15–17]. Although these factors have been implicated in supporting spermatogenesis, it remains unclear which specific factors are essential in testicular cellconditioned medium (TCCM) for driving PGC differentiation *in vitro*. While neonatal testicular cell cultures suggest that multiple somatic and germ cells are likely to contribute paracrine signals, there is yet no definitive mapping of exactly which cell types produce which signals required for efficient PGC development.

Culturing testicular cells from 5-day-old mice for 6 days revealed various cell types likely responsible for producing the growth factors necessary for *in vitro* spermatogenesis. Other studies have also explored the use of conditioned medium from 1-day-old mouse testicular cells [15], rat [16], and testicular sperm extraction [18] to induce embryonic stem cells to differentiate into gamete cells. This study investigated the ability of testicular cell-conditioned medium produced from neonatal mouse testicular cell cultures to induce *in vitro* differentiation of male PGCs *in vitro* and to identify secreted proteins present in the conditioned medium responsible for promoting PGC differentiation.

## Materials and Methods

### Ethical approval

The animals used in this study were approved by the Animal Ethics Commission at the School of Veterinary Medicine and Biomedical Sciences, IPB University, under the certificate number 089/KEH/SKE/III/2018.

### Animals

Primordial germ cells were harvested from mouse fetuses approximately 13.5 days postcoital (dpc)/E 13.5, by mating mice in a 1:1 ratio without hormonal stimulation. The following day, vaginal observations were conducted; the presence of a vaginal plug indicated H1 postcoitus.

### Collection of conditioned medium

Testicular cells from 5-day-old mice were cultured for 6 days; on this day, the cell population was above 90% confluence [19]. TCCM was collected in serum-free DMEM [20] to avoid protein contamination. The conditioned medium was collected, filtered through 0.22 μm pores, and stored at −20°C for further use.

### Proteomic analysis

Proteomic analysis in this research involved determining protein concentration, enrichment with ProteoMiner, sample preparation via the filter-aided sample preparation method, peptide clean-up, protein profiling using liquid chromatography–tandem mass spectrometry (LC-MS/MS), and data processing with Proteome Discoverer 2.1.

### Germ cell culture and treatments

Germ cells were isolated from the genital ridge using the method developed by Moreno-Ortiz et al. [21]. The isolated germ cells were cultured in DMEM supplemented with 15% FCS and 1,000 IU/ml LIF (Sigma) for six days to enhance the *in vitro* proliferation of PGCs. The viability of germinal cells at day-0 and day-6 was 87% and 89.4%, respectively [12]. The PGC were divided into three groups: (a) control group medium (DMEM with 15% FCS), (b) 40% TCCM and 60% DMEM+15% FCS, and (c) 60% TCCM and 40% DMEM+15% FCS. Germ cells were cultured for 4 weeks at 37°C and 5% CO₂ in air [15,18]. The cells were collected and dissociated using 0.1% trypsin in DPBS at 37°C for 10 min. Repeated pipetting was performed during the dissociation process to accelerate the separation. Completely dissociated cells were centrifuged for 4 min at 3,000 × gm, and the supernatant was discarded to obtain cell pellets. These pellets were preserved for gene expression analysis using reverse transcription-polymerase chain reaction (RT-PCR).

### Real-time PCR

Following the specified protocol, RNA was isolated using the RNeasy Mini Kit (Cat No./ID: 74106). RNA concentration was measured using a NanoDrop^™^ One Microvolume UV-Vis Spectrophotometer (Thermo Scientific) at 260/280 and 260/230 nm. Complementary deoxyribonucleic acid (cDNA) was synthesized using the SensiFAST kit (Bioline^®^, Bio-65054), according to the manufacturer’s instructions. Quantitative real-time PCR (qPCR) was performed using the Bio-Rad iQ5 Multicolor Real-Time PCR system. The RT-PCR reaction mixture consisted of 20 μl, containing 1 μl each of forward (F) and reverse (R) primers, 2 μl of reverse cDNA, 10 μl of Sensifast SYBR Green, and 6 μl of DNase/RNase-free water. The qPCR conditions were as follows: 95°C for 2 min, followed by 40 cycles of 15 sec at 95°C, 20 sec at either 56°C or 50°C, and 10 sec at 65°C. Eight genes were analyzed using actin as the housekeeping gene [22–24]. The expression of pluripotency (*Oct*4), PGCs (*Dazl*), peri-meiotic gonocytes (*Vasa*), meiosis (*Stra8)*, post-meiosis (*Prm1*), mature sperm (*Acr*), oogenesis (*Gdf9*), and housekeeping gene (Actin) (Table 1).

**Table 1.** The primers used for PGC identification.

Gene	Forward Primer	Reverse Primer	References
*Oct4*	5’-GCAGATCACTCACATCGCCA-3’	5’-CACCCGAGGCTCAAGCTTC-3’	[29]
*Mvh/Vasa*	5’-TATGATGCGGGATGGAAT-3’	5’-CACCCTTGTACTATCTGTCG-3’	[29]
*Dazl*	5’-AATGTTCAGTTCATGATGCTGCTC-3’	5’-TGTATGCTTCGGTCCACAGACT-3’	[29]
*Stra8*	5’-GTTTCCTGCGTGTTCCACAAG-3’	5’-GTTTCCTGCGTGTTCCACAAG-3’	[29]
Protamine* (Prm1)*	5’- CCAGCACCAT GGCCAGATAC-3’	5’- GATGTGGCGAGAT GCTCTTG-3’	[30]
*Acr*	5’-GAAACAAGCCAGTGAAAGA-3’	5’-CAGCAGGGTCCAATGAAG-3’	[29]
*Gdf-9*	5’-CATGGGGGCCACTTCAACAA-3’	5’-TGGGGAGAAAGAGCTCTCCAA-3’	[30]
*Actin*	5’-AGAGGGAAATCGTGCGTGAC-3’	5’-CAATAGTGATGACCTGGCCG-3’	[29]

### Data analysis

The growth characteristics of germ cells were qualitatively described after *in vitro* culturing. Relative gene expression was analyzed using one-way ANOVA with a confidence level greater than 95%. Data from the LC-MS/MS were analyzed using Proteome Discoverer 2.1 software for database-based protein identification. Within this software, the Sequest HT search engine was employed in conjunction with the Mus musculus database (Taxonomy ID: 10090), sourced from www.uniprot.org. Trypsin was used, allowing for a maximum of two missed cleavages. The permitted modifications included oxidation of methionine (Met), acetylation of the N-terminus, and carbamidomethylation of cysteine. Functional analysis of the identified proteins was conducted using the DAVID version 6.8 website (https://david.ncifcrf.gov), whereas pathway-related protein analysis was performed using the KEGG database (https://kegg.jp). Protein interactions were assessed using STRING version 11.0 (https://string-db.org).

## Results

### Proteomic profile of TCCM

**Figure 1.**
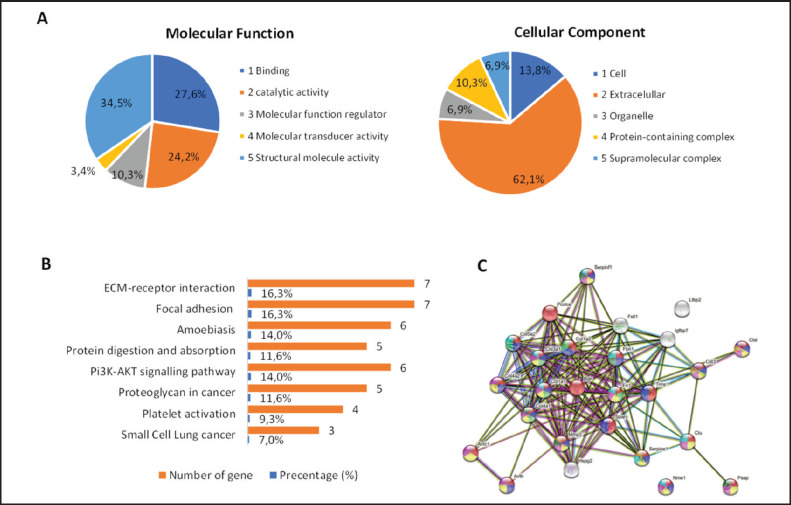
Proteomic analysis of conditioned medium (CM) of testicular cell culture of 5-day-old mice. (A) GO analysis of CM on molecular function and cellular components (http://www.pantherdb.). (B) Pathway based on protein contained in CM (https://david.ncifcrf.gov). (C) Protein interaction by using STRING analysis (https://string-db.org).

The LC-MS/MS data processing results obtained using Proteome Discoverer 2.1 software with the Mus musculus database (Taxonomy ID: 10090) identified 26 proteins (Table 2). Only proteins with a minimum of two unique peptides were analyzed to minimize misidentification [25]. Based on the identified protein accession numbers, gene ontological (GO) analysis using the PANTHER Classification System shows that the proteins in the TCCM in molecular function (Fig. 1A) play a role in the process of “binding (27.6%)”, “catalytic activity (24.2%)”, “molecular function regulator (10.3%)”, “molecular transduction activity (3.4%)”, and ‘molecular structure activity (34.5%)’. The protein in CM with the highest percentage (34.5%) plays a role in ‘molecular structure activity. Proteins that play a role in the activity of molecular structures are collagen 1(I), 1(III), 1(IV), 2(I), 2(IV), 2(V), Fibrinillin-1, Actin, heart muscle alpha 1, cytoplasmic 1, and latent transforming growth factor beta-binding protein 2. As cell components, most proteins played a role in the extracellular component (62.1%) and the least in supramolecular proteins (6.9%). DAVID analysis revealed a set of proteins that regulate cell pathways, a finding that adds a new dimension to our understanding of molecular biology. Among the eight identified pathways (Fig. 1B), three were particularly interesting: extracellular matrix (ECM)-receptor interaction, focal adhesion, and the PI3K-AKT signaling pathway. The proteins involved in these pathways are five collagen proteins (COL1A1, COL1A2, COL3A1, COL4A1, AND COL5A2) found in CM, each with slight protein differences, a finding that piques our curiosity and prompts further investigation. STRING analysis demonstrated the versatility of the 26 identified proteins (Fig. 1C), each of which is capable of playing a role in multiple biological processes (Table 3).Notably, however, key soluble regulators of spermatogenesis frequently reported in the literature (e.g., BMP4, SCF, LIF, and GDNF) were not detected in our dataset, suggesting that the contribution of TCCM in this study may be primarily mediated through ECM-derived components rather than classical growth factors.

**Table 2.** Proteins identified in the conditioned medium of a 5-day mouse testicular cell culture based on the Mus musculus database.

Name of Protein	Number of Accessions	Number of unique peptides	Score sequest (HT > 0)	Location subcellular	Function
The Extracellular matrix:					
Collagen alpha-1(I) chain (COL1A1)	P11,087-1	34	193	Extracellular matrix	Structure, extracellular matrix, binding
Collagen alpha-2(I) chain COL1A2	Q01,149	29	198	Extracellular matrix	Structure, extracellular matrix, binding
Collagen alpha-2(V) chain (COL5A2)	Q3U962	3	15	Extracellular matrix	A key determinant in the assembly of tissue-specific matrices
Collagen alpha-1(III) chain (COL3A1)	P08,121	3	14	Extracellular matrix	Extracellular matrix
Fibronectin (FN1)	P11,276	11	43.8	Extracellular matrix	Cell Adhesion, signaling-receptor binding,
Biglycan (BGN)	P28,653	3	17	Extracellular matrix	Collagen fiber assembly, extracellular matrix binding
Fibrillin-1 (FBN1)	Q61,554	3	12	Extracellular matrix	Components of extracellular matrix structure
Latent-transforming growth factor beta-binding protein 2 (LTBP2)	O08,999	2	5	Extracellular matrix	Plays a role in the organization of fiber-elastic structures
Actin, alpha cardiac muscle 1 (ACTC1)	P68,033	2	33	Extracellular matrix	Actin-filament organization
Cell Structure:					
Collagen alpha-1(IV) chain (COL4A1)	P02,463	11	77	Basement membrane	the major structural component of basement membranes
Collagen alpha-2(IV) chain (Col4a2)	P08,122	2	8	Basement membrane	the major structural component of basement membranes
SPARC (SPARC)	P07,214	11	101	Basement membrane	regulate cell growth through interactions with the extracellular matrix and cytokines.
Basement membrane-specific heparan sulphate proteoglycan core protein (HSPG2)	Q05,793	2	7	Basement membrane	Component of the basement membrane
Cathepsin L1 (CTSL)	P06,797	3	5	Basement membrane, Lysosome	Thiol protease important for the overall degradation of proteins in lysosomes
Actin, cytoplasmic 1 (ACTB)	P60,710	7	82.3	Cytoskeleton, Nucleus	Actin is a highly conserved protein that polymerizes to produce filaments that form cross-linked networks in the cytoplasm of cells. Regulate gene transcription and motility and repair of damaged DNA
72 kDa type IV collagenase (MMP2)	P33,434-1	2	6	extracellular space, mitochondrion, nucleus, plasma membrane	Various functions include vascular remodeling, angiogenesis, tissue repair, tumors invasion, and inflammation.
Nucleoside diphosphate kinase A (NME1)	P15,532	2	4	Nucleus, cytoplasm	Key role in the synthesis of nucleoside triphosphates other than ATP
Secretion:					
Plasminogen activator inhibitor 1 (SERPINE1)	P22,777	17	127	Secretion	Protease binding, Signalling-receptor binding, Serine protease inhibitor
Clusterin (CLU)	Q06,890	12	45	Secreted, nucleus, cytoplasmic, cytosol, mitochondria	Binding regulation the cell proliferation
Insulin-like growth factor-binding protein 7 (IGFBP7)	Q61,581	8	39	Secreted	Binds IGF-I and IGF-II with a relatively low affinity Stimulates prostacyclin (PGI2) production. Stimulates cell adhesion
Follistatin-related protein 1 (FSTL1)	Q62,356	8	4	Secreted	Secreted glycoprotein that is involved in various physiological processes, such as angiogenesis, regulation of the immune response, cell proliferation and differentiation
Metalloproteinase inhibitor 1 (TIMP1)	P12,032	7	29.7	Secreted	Growth factor that regulates cell differentiation, migration and cell death and activates cellular signaling cascades via CD63 and ITGB1. Plays a role in integrin signaling
Pigment epithelium-derived factor (SERPINF1)	P97,298	6	26.3	Secreted, melanosome	Neurotrophic protein; induces extensive neuronal differentiation in retinoblastoma cells.
Cystatin-C (CST3)	P21,460	5	34	Secreted	This protein is thought to serve an important physiological role as a local regulator of this enzyme activity
Procollagen C-endopeptidase enhancer 1 (PCOLCE)	Q61,398	4	13	Secreted	Binds to the C-terminal propeptide of type I procollagen and enhances procollagen C-proteinase activity.
Prosaposin (PSAP)	Q61,207	2	8	Secreted, Lysosome	Behaves as a myelinotrophic and neurotrophic factor, these effects are mediated by its G-protein-coupled receptors, GPR37 and GPR37L1, undergoing ligand-mediated internalization followed by ERK phosphorylation signaling

**Table 3.** Biological processes of 26 proteins that were identified using STRING analysis. The colored circle corresponds to the color of the STRING analysis result.

No ID	Biological process	Number of proteins	False discovery rate	Proteins
GO:0010033	Response to organic substances	22	3.29e–14	SERPIN1, PCOLCE, COL5A2, COL4A2, COL1A2, COL3A1, COL1A1, COL4A1, BGN, FBN1, FN1, SPARC, MMP2, ACTC1, ACTB, SERPINF1, NME1, PSAP, CLU, TEMP1, CST3, CTSL
GO:0009719	Response to endogen stimulus	16	2.64e–11	ACTB, NME1, MMP2, COL4A2, COL1A1, COL3A1, COL5A2, SPARC, SERPINE, SERPINEF1, COL1A2, FBN1, TEMP1, CST3, CTSL
GO:0030198	Extracellular matrix	6	2.74e–06	COL5A2, COL3A1, COL1A2, COL1A1, COL4A1, FN1
GO:0048869	Process of developing cells	15	4.20e–05	SERPINF1, COL3A1, COL4A2, COL1A1, COL4A1, ACTC 1, MMP2, FN1, SERPINE, CLU, CST3, CTSL, ACTB, NME1, PSAP
GO:0030154	Cells differentiation	14	0.00016	SERPINF1, COL4A2, COL1A1, COL4A1, COL3A1, ACTC 1, MMP2, CLU, CST3, CTSL, FN1, ACTB, NME1, PSAP
GO:0001655	Development of the urogenital system	5	0.00061	COL4A1, MMP2, FBN1, PSAP, SERPINEF1
GO:0045595	Regulations of cell differentiation	9	0.0014	NME1, SERPINE, CLU, FN1, COL1A1, COL3A1, FBN1, COL5A2, SERPINF1
GO:0008406	Gonad development	4	0.0016	CST3, CTSL, SERPINEF1, MMP2
GO:0060008	Differentiation of sertoli cells	2	0.0045	CST3, CTSL

### Germ cell culture and TCCM induction

**Figure 2.**
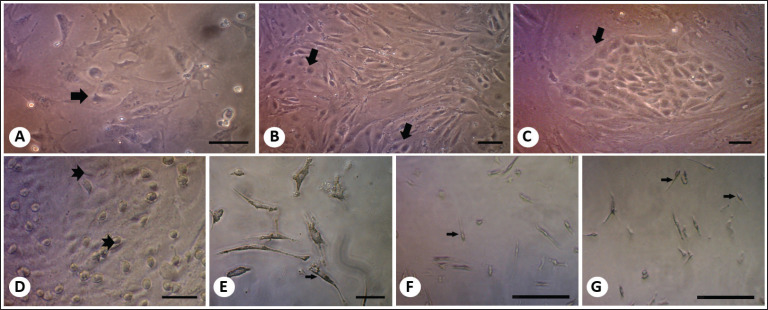
Characteristics of germinal cells cultured *in vitro*.Top row photo(A), Day 3. (B) Day 6. (C) Day 14. Germinal cells that are about to start colonizing (arrow). Bar = 50 µm. Down-row Photo: (D) Day 18, round-shaped spermatid-like(arrow). (E-G) Day 24, tailed sperm-like structures(arrow) in the control group (E), group CM 40% (F), and group CM 60% (G). Bar = 100 µm.

Cells were cultured from the entire genital ridge, which was separated from the mesonephric ducts. Histological analysis of the genital ridge revealed two cell types within the testicular cord: germ cells PGCs, and Sertoli cells. Initially, the culture of the entire genital ridge displayed solitary cells; however, by the third day, 2–3 germ cells began to cluster. By the sixth day, these cells had started to form small colonies. By day 14, the number of colonized germ cells had increased, exhibiting structures resembling testicular cords. After 18 days of culture, round spermatid-like cells were observed; by day 24, cells resembling sperm-like structures were present (Fig. 2). However, as no quantitative evaluation of differentiation efficiency was performed, these findings should be interpreted as qualitative observations rather than definitive evidence of enhanced differentiation.

### Gene expression using real-time PCR

**Figure 3.**
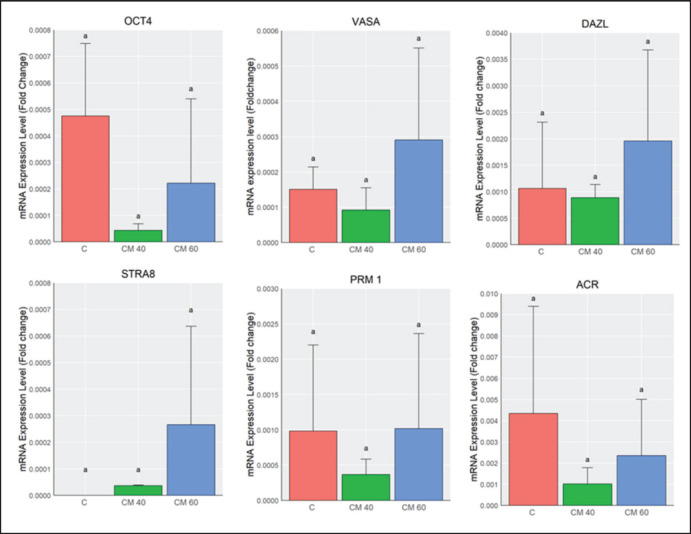
Relative expression of the marker genes involved in spermatogenesis. The relative expressions of *Oct4*, *Dazl*, *Vasa*, *Stra8*, *Prm1*, and *Acr* were quantified by RT-PCR in cells cultured with 40% TCCM, 60% TCCM, and control.

The analysis of PGC development, *in vitro* gametogenesis, and gene expression, as determined using RT-PCR in the treatment group, is presented in Figure 3. Germinal cell cultures treated with TCCM at 40% and 60% and the control showed distinct induction patterns. RT-PCR analysis revealed that the *Oct4*, *Dazl*, *Vasa*, *Stra8*, *Prm1*, and *Acr* genes were expressed in each group, with no statistically significant differences (*p* > 0.05). *Oct4*, *Dazl*, *Vasa*, *Stra8*, *Prm1*, and *Acr* were expressed in each group. Although the differences among groups were not significant (*p* > 0.05), a trend of higher expression of meiosis- and post-meiosis-related markers (*Stra8, Prm1,* and *Acr*) was observed in the 60% TCCM group compared to the 40% TCCM group. In contrast, the pluripotency marker *Oct4* was relatively higher in the control, suggesting the persistence of undifferentiated cells. The *Gdf9* gene, which is essential for folliculogenesis, was not expressed; *Gdf9* mRNA is produced exclusively in oocytes from the primary stage to ovulation [26]. This analysis aimed to confirm the transition of germ cells from male PGCs to oocyte-like cells with the suppression of *Gdf9*, suggesting that the induction was effective.

## Discussion

The profiling of proteins in the secretome TCCM, primarily comprising structural proteins that form the ECM and cell membrane components, plays a significant role in our understanding of testicular cell biology. Some proteins, such as ﬁbronectin, collagens I, III, IV, and V, SPARC, and fibronectin-1, are similar to those identified in human peritubular cells [27]. Flenkenthaler et al. [27]stated that CM from human testicular peritubular cells (HTPCs) contributes to the formation of the basement membrane and structure of the tubule wall, thereby forming the SSC niche along the tubule. TCCM, like HTPCs, provides valuable insights, especially considering the presence of many somatic cells and myoid cells in culture; however, no specific proteins that support spermatogenesis were identified in TCCM.

The extracellular matrix primarily consists of glycoproteins and polysaccharides that fill the extracellular space between cells and other related cells. In rodent testes, the ECM has a unique shape and consists primarily of collagen, type IV laminin, and heparan sulfate proteoglycan lining each seminiferous tubule [26,28]. The extracellular matrix and basement membrane are associated with Sertoli cells and spermatogonia, respectively. In addition to Sertoli cells and spermatogonia, the blood-testis barrier (BTB) is also attached to the basement membrane. The blood-testis barrier physically divides the seminiferous epithelium into basal and abluminal (apical) parts, a division that occurs due to the development and maturation of germ cells [28].

**Figure 4.**
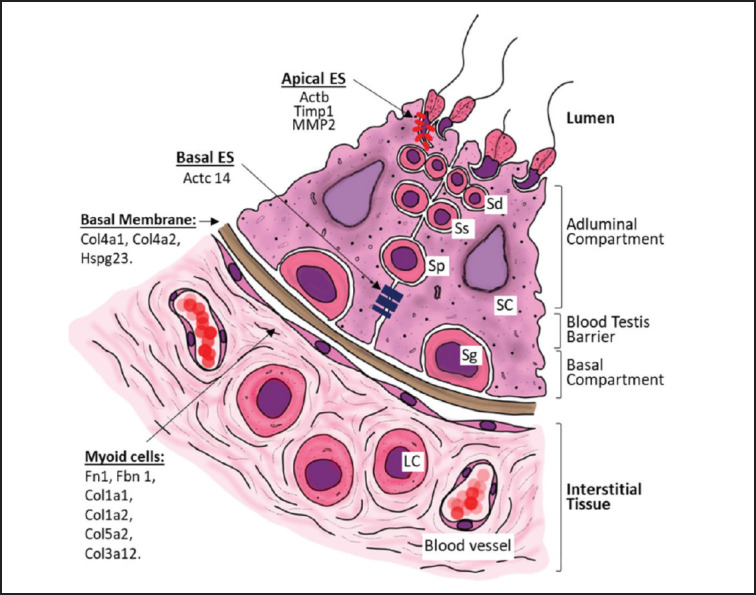
Schematic of spermatogenesis involving proteins in the CM.

Under* in vitro* conditions, proteins in the TCCM of testicular cells from 5-day-old mice cultured for 6 days formed an extracellular matrix that resembled the* in vivo* conditions (Fig. 4). *In vitro*, the extracellular matrix acts as a reservoir for growth factors and forms BTB-like compartments. Under *in vivo* conditions, actin in basal ectoplasmic specialization (ES) plays a role in the movement of spermatogonia towards the lumen. In contrast, actin in the apical ES plays a role in spermiation [28]. The presence of MMP2 and TIMP1 plays a role in ECM remodeling, which is involved in the development process, tumor invasion, metastasis, cell movement, and the Sertoli cell tight junction barrier [26]. MMP2 and TIMP1 proteins are detected when spermatids detach from the epithelium, a process known as spermiation. Under *in vitro* conditions, the presence of MMP2, TIMP1, and actin in the apical part of the ES causes sperm-like structures to migrate out of the cell colony into the space in the petri dish. MMP2 and TIMP1 play a role in ECM remodeling by proteolyzing laminin to form functional laminin fragments that facilitate spermatid movement and spermiation. In this study, RT-PCR analysis of germ cell markers (*Oct4*, *Dazl*, *Vasa*, *Stra8*, *Prm1*, and *Acr*) showed no statistically significant differences between the control and TCCM-treated groups. This outcome is likely influenced by the limited sample size (duplo), which contributes to variability in the data. Moreover, the impact of TCCM on germ cell differentiation may not be solely reflected at the transcriptional level but rather through paracrine signaling and interactions with somatic cells, particularly Sertoli cells, that establish the testicular niche. Under *in vitro* conditions, TCCM and Sertoli cells of the genital ridge provide structural and nutritional support, regulate the microenvironment, and maintain specialized compartments essential for the development of germ cells. Known as the “nurse cells,” the Sertoli cells provide essential nutrients for developing germ cells and create specialized compartments within the seminiferous tubules through tight junctions.

The gene marker expression for germ cells for understanding the differentiation pathway stimulated by TCCM. The expression of *Oct4* in the control group indicates that, without inductive cues, some cells retained a pluripotent or undifferentiated state. The cultures were supplemented with TCCM at a concentration of 60%, which resulted in increased levels of *Stra8*, *Prm1*, and *Acr*. Gene expression indicates meiotic initiation and is associated with haploid germ cells (Fig. 3). Although the differences did not reach significance, the upward trend observed in the 60% group suggests that higher concentrations of conditioned medium may create an environment conducive to meiotic entry. *Stra8* is recognized as a critical marker for the onset of meiosis, whereas *Prm1* and *Acr* are characteristic of post-meiotic development [29,30]. The absence of *Gdf9* expression in our cultures also aligns with earlier reports, since this gene is expressed only in female oocytes during folliculogenesis [26]. These compartments provide a unique environment for germ cell development. Sertoli cells also regulate spermiation, secretion of fluid, proteins, and various growth factors, and phagocytize degenerated sperm.

Although our molecular results remain inconclusive, these cellular interactions still suggest a potential role of TCCM in promoting germ cell maturation. The entire genital ridge (crude genital ridge) was cultured to obtain PGCs without the need for PGC purification. On day 14 of culture, PGCs colonized, and other cells provided support. Sertoli cells and other somatic cells play a crucial role in cell development, which is thought to act as a feeder layer, allowing PGCs to develop properly. The success of our research was evidenced by the appearance of spermatid-like structures on day 18 of culture and sperm-like structures with tailed structures and oval heads on day 24. These promising results indicate the potential application of our findings in the field of reproductive biology. The development of germ cells, apart from morphological changes, is characterized by the expression of marker genes at the PGC, pre-meiosis, and post-meiosis stages. In our study, the expression of meiosis-related genes, such as *Stra8*, *Prm1*, and *Acr*, suggests that some germ cells had initiated meiotic progression. However, we acknowledge that gene expression analysis alone does not provide direct confirmation of haploid DNA content.

Therefore, no specific proteins in the TCCM were identified; however, proteins that function as the ECM were detected. This protein, as an ECM, showed promising results in supporting spermatogenesis *in vitro*, indicating that our findings have the potential to be applied in the field of reproductive biology.

## Conclusion

TCCM proteins and Sertoli cells contribute to the development of seminiferous tubule-like structures that closely mimic the *in vivo* conditions. Notable advancements were observed in germ cell culture experiments, with cell colonies forming testicular cord-like structures by Day 14. By day 18, spermatid-like cells had emerged, followed by the appearance of sperm-like structures on day 24.

## List of abbreviations

ART, assisted reproductive technology; BMP4, bone morphogenetic protein 4; bFGF, basic fibroblast growth factor; CM, conditioned medium; cDNA, complementary deoxyribonucleic acid; DMEM, Dulbecco’s Modified Eagle Medium; ECM, extracellular matrix; ES, ectoplasmic specialization; GDNF, glial cell-derived neurotrophic factor; FCS, fetal calf serum; LC-MS/MS, chromatography–mass spectrometry; LIF, leukemia inhibitory factor; PCR, Polimerase Chain Reaction; PGC, Primordial Germ Cells; RT-PCR, reverse transcription-polymerase chain reaction; SCF, stem cell factor; SSC, spermatogonial stem cell; TCCM, testicular cell conditioned medium; TESE, testicular sperm extraction; RA, retinoic acid.
